# *Brucella melitensis* clinical isolate modulates osteoclast differentiation to drive pathological bone destruction in brucellar arthritis

**DOI:** 10.3389/fcimb.2025.1694633

**Published:** 2025-12-12

**Authors:** Jinlei Chen, Feijie Zhi, Guanghai Zhao, Mengru Su, Junyun Tuo, Wei Song, Yuefeng Chu, Haihong Zhang

**Affiliations:** 1 Orthopedics Key Laboratory of Gansu Province, Department of Orthopedics, The Cuiying Biomedical Research Center, The Second Hospital & Clinical Medical School, Lanzhou University, Lanzhou, China; 2 State Key Laboratory for Animal Disease Control and Prevention, College of Veterinary Medicine, Lanzhou University, Lanzhou Veterinary Research Institute, Chinese Academy of Agricultural Sciences, Lanzhou, China; 3 Gansu Province Research Center for Basic Disciplines of Pathogen Biology, Lanzhou, China; 4 Key Laboratory of Veterinary Etiological Biology, Key Laboratory of Ruminant Disease Prevention and Control (West), Ministry of Agricultural and Rural Affairs, Lanzhou, China

**Keywords:** *B. melitensis* biovar 3, bone resorption, brucellar arthritis, osteoclast differentiation and function, pathological bone destruction

## Abstract

**Background:**

Brucellosis is a widespread zoonosis that is acquired by humans from infected animals. Articular complications, particularly brucellar spondylitis, are the most prevalent and disabling manifestations of human brucellosis. Inflammation-mediated osteoclast activation is implicated in *Brucella*-induced bone destruction, but the direct cellular tropism of *Brucella* within bone tissue and the specific effects of infection on osteoclasts remain poorly understood. This study aims to characterize the osteoclast tropism of *Brucella melitensis* biovar 3 clinical isolates and their direct regulatory effects on osteoclast-mediated bone destruction in *Brucella*-induced arthritis.

**Methods:**

*Brucella* clinical isolates were obtained from the bone tissues of human brucellar spondylitis patients in Gansu Province, China. Whole-genome sequencing and biotyping identified their specific biovars. These isolates were used to generate arthritis in immunodeficient NCG mice; bone homeostasis in these mice was assessed via ELISA. We assessed their cellular tropism and osteoclast-modulating effects through intracellular survival assays, immunofluorescence, histopathology, TRAP staining, and resorption pit analysis.

**Results:**

Three clinical isolates of *B. melitensis* biovar 3 were obtained from arthritis lesions in patients from Gansu. Genomic analysis revealed homology with geographically diverse Chinese *Brucella* strains. Although these isolates reached splenic bacterial loads similar to the virulent strain 16M, they did not cause splenomegaly by two weeks post-infection. The isolates displayed strong tropism for human and murine osteoclasts, achieving significantly higher intracellular loads compared to osteoblasts or osteocytes. Infection at the osteoclast precursor/bone marrow macrophage stage enhanced early osteoclastogenesis while inhibiting late-stage apoptosis and fusion, leading to prolonged osteoclast survival and aggravated bone resorption and defects. In contrast, conditioned medium from infected osteoblasts or osteocytes had minimal impact on late-stage osteoclast differentiation.

**Conclusions:**

These findings elucidate the mechanisms underlying pathological bone defects in brucellar arthritis. The direct bacterial effects, together with the formation of an osteoclast-derived pro-survival niche, account for the prevalence of brucellar arthritis as the most common complication of chronic brucellosis. Targeting the interaction between *B. melitensis* and osteoclasts may thus offer a novel therapeutic strategy for preventing and treating *Brucella*-induced osteolytic lesions.

## 1 Introduction

Brucellosis, first described clinically in the 1859 Malta outbreak by Marston, was confirmed etiologically when David Bruce isolated *Brucella* spp. from human splenic tissue in 1886 (Aleixo et al., 1999). This zoonosis poses dual threats to global public health and livestock economies, particularly in developing regions (Maurin, 2005), yet remains underprioritized despite the significant burden that the disease imposes (Laine et al., 2023). Current estimates indicate that 2.1 million human cases occur annually which surpasses prior projections and justifies WHO (World Health Organization) classification of the disease as a neglected zoonosis (Bundle and McGiven, 2017; Khurana et al., 2021). Transmission of the bacterium occurs through interconnected pathways: livestock acquire infection via contaminated feed, water or abortigenic materials, whereas humans are infected through direct or indirect contact, environmental exposure, or unpasteurized dairy consumption (Xingxing et al., 2024; Wen et al., 2024). Africa and Asia bear the highest disease burden, with China reporting an 8.20% annual incidence increase between 2004 and 2021 (Bundle and McGiven, 2017; Cai et al., 2019). Brucellosis imposes substantial burdens on both livestock industry development and public health in developing countries. Although rarely fatal, human brucellosis causes severe debilitation and brucellar spondylitis represents a leading cause of morbidity and mortality (Wang and Zhang, 2022). Acute cases present with non-specific influenza-like symptoms (Spernovasilis et al., 2024), whereas chronic manifestations include arthritis, reproductive inflammation, endocarditis, and meningitis (Wang et al., 2023). First-line antibiotic therapy fails to prevent relapse and chronic complications in certain patients (Qureshi et al., 2023; Aydin et al., 2005). However, brucellar arthritis, which is the most prevalent chronic complication of brucellosis, remains understudied due to multiple limiting factors.

Brucellar osteoarticular complications affect up to 85% of patients (Arkun and Mete, 2011; Gotuzzo et al., 1982; Khateeb et al., 1990) and manifest as sacroiliitis, spondylitis, osteomyelitis, or peripheral arthritis (Alamian et al., 2021; Bosilkovski et al., 2016; Tali et al., 2015). Age determines involvement patterns: children develop monoarticular arthritis in knees or hips (Bosilkovski et al., 2013; Esmaeilnejad-Ganji and Esmaeilnejad-Ganji, 2019), whereas adults exhibit sacroiliac (80%) and spinal (54%) involvement (Jeyaraman et al., 2023). Brucellar spondylitis carries the gravest prognosis (Ma et al., 2022), with histopathology revealing granulomatous infiltration, osteonecrosis, and osseous defects that cause spinal instability (Skyberg et al., 2012). Identifying the mechanisms that underlie *Brucella*-induced pathological bone defects is critically important, as it may help to reduce disability rates in brucellar spondylitis. The mechanisms of brucellar arthritis bone defects remain poorly characterized, but inflammation may be implicated: *Brucella* invades osteoblasts, osteocytes, and immune cells which triggers inflammatory factors and matrix metalloproteinases that promote osteoclast differentiation (Delpino et al., 2012, 2009; Giambartolomei et al., 2012; Hu et al., 2020). Alternatively, suppressed osteoblast function may contribute to pathology (Pesce Viglietti et al., 2018; Gentilini et al., 2018; Freiberger et al., 2023), although direct experimental evidence for *Brucella*-osteoclast interactions remains limited. Crucially, the single study that examined these interactions focused exclusively on *B. abortus* (Khalaf et al., 2020), despite epidemiological evidence that >84.5% of human brucellosis cases in China, including arthritis complications, are caused by *B. melitensis* (Pesce Viglietti et al., 2016; Zhu et al., 2020). Therefore, experimental investigation using clinically-isolated *Brucella* strains are both scientifically imperative and methodologically essential to validate the potential tissue-specific tropism of *B. melitensis* and to assess the impact of this species on functioning of human osseous tissues and cells.

Spinal tropism of *Brucella* may stem from vertebral arterial vasculature density (Ratcliffe, 1985) that enables hematogenous spread to medullary cavities with subsequent dissemination (Tali et al., 2015; Morales, 2018). Nevertheless, systematic studies on cellular tropism of the bacterium within bone are lacking. Preferential colonization of specific bone cell types likely facilitates persistence and osteoarticular complications. Critically, it remains unknown whether clinical isolates of *Brucella* directly target osteoclasts and whether these isolates exert specific effects on osteoclast differentiation and fusion to promote pathological bone loss which is a factor that may explain the greater propensity of *B. melitensis* than *B. abortus* in causing arthritis. Here, we isolated *B. melitensis* biovar 3 from bone lesions of brucellar arthritis patients. These isolates showed *B. melitensis* 16M-comparable virulence, bone colonization capacity, and marked osteoclast tropism. The strains exerted biphasic regulation on osteoclast differentiation, promoting early differentiation while suppressing late-stage apoptosis and fusion that culminated in osteoclast accumulation and pathological defects. We conclude that this biphasic modulation, combined with the osteoclast survival niche, constitutes a key mechanism of bone destruction by *B. melitensis* in human brucellar arthritis.

## 2 Materials and methods

### 2.1 Study subjects

Three patients with brucellar arthritis undergoing spinal surgery were enrolled at the Second Hospital of Lanzhou University, China between October 2023 and March 2025. Diagnosis required clinical brucellosis manifestations with significant specific antibody titers (standard tube agglutination test ≥1:100), and isolation of *Brucella* from affected bone tissue. Demographic characteristics and computed tomography and magnetic resonance imaging findings are presented.

### 2.2 *Brucella* strains

*Brucella* strains were isolated from surgically collected bone tissue specimens using standard bacteriological methods in Animal Biosafety Level 3 Laboratory (ABSL-3) facilities. Briefly, specimens were inoculated into biphasic blood culture bottles supplemented with 5-10% horse serum and were incubated at 37 °C under continuous agitation at 180 rpm on an orbital shaker for a minimum of one week. Turbid cultures were streaked onto tryptic soy agar (TSA) for colony isolation. Candidate colonies underwent species identification via 16S rRNA, BCSP31, and AMOS-PCR assays that are used to differentiate *Brucella* species. Biotyping assessed CO_2_ requirement, H_2_S production, growth on thionin and basic fuchsin selective dyes, and agglutination with monospecific antisera (A/M/R) (Al Dahouk et al., 2003; Yagupsky et al., 2019).

Strains used included virulent *B. melitensis* 16M (CVCC789) reference strain, vaccine strain Rev.1 (CVCC790), and three clinical *Brucella* isolates obtained from human bone specimens. Bacteria were cultured on TSA at 37 °C with 5% CO_2_. Single colonies were inoculated into Tryptic Soy Broth with gentamicin (50 μg/mL bactericidal; 25 μg/mL maintenance) and grown to OD_600_ ~0.6. All work with live pathogens occurred in BSL-3 facilities (Zhi et al., 2024).

### 2.3 Whole genome sequencing

Bacterial suspensions were heat-inactivated, genomic DNA was extracted using the TIANGEN Bacterial DNA Kit (Beijing, China) and DNA quality was assessed. Libraries were prepared by DNA fragmentation, end repair, and adapter ligation, and whole genome sequencing was performed on the PacBio Sequel II platform. Raw data were processed using SMRT Link 11.0.0. The genome has been deposited in NCBI under BioProject PRJNA1357550.

### 2.4 Phylogenetic analysis

Whole-genome single nucleotide polymorphisms (wgSNPs) between clinical isolates and publicly available *B. melitensis* strains from NCBI were analyzed using Snippy (v4.6.0), followed by recombination SNP removal with Gubbins (v3.4). IQ-TREE2 (https://github.com/iqtree/iqtree2) was employed to identify the best-fit substitution model based on wgSNPs, with the model showing the lowest Bayesian Information Criterion value being selected. Maximum likelihood phylogenetic trees subsequently were constructed using IQ-TREE2 under the optimal model with 1000 bootstrap replicates. Final visualization was performed using the Interactive Tree Of Life platform (https://itol.embl.de/).

### 2.5 Animal experiments

Female immunodeficient NOD-Prkdcem26Il2rgem26/Gpt (NCG) mice (6–8 weeks; GemPharmatech Co., Ltd., Chengdu, China) and immunocompetent age-matched female BALB/c mice (Lanzhou Veterinary Research Institute, CAAS, Lanzhou, China) were used (Khalaf et al., 2019). Animals were acclimatized for seven days in BSL-3 certified individually ventilated cage systems. For infection studies, NCG mice received intraperitoneal injections of 0.1 mL containing 1×10^4^ or 1×10^5^ CFU of the test bacterium; BALB/c mice received injections of 1×10^5^ CFU. Spleen and bone samples were collected at predetermined time points post-infection for subsequent experimental analyses. C57BL/6 mice (Lanzhou Veterinary Research Institute, CAAS) were used to isolate primary bone marrow-derived monocytes (BMMs). All procedures were approved by the Lanzhou Veterinary Research Institute Ethics Committee (LVRIAEC-2024-096).

### 2.6 ELISA assay

Mouse blood was obtained from the Fundus vein. Samples were allowed to clot for 2 h at room temperature and then centrifuged for 15 min at 12,000 rpm at 4 °C. Serum was collected and frozen at −20 °C until use. OPG (Catalog # RK04789) and RANKL (Catalog # RK00149) ELISA kits were purchased from ABclonal. Serum OPG and RANKL produced *in vivo* by NCG PBS and NCG 10^5^ mice were measured by ELISA assay according to the manufacturer’s instructions. All the optical densities (ODs) measured after reactions were converted to the concentration using their standard curves. All the samples were measured in triplicate.

### 2.7 Cell culture

Primary BMMs were isolated from C57BL/6 mice femurs and tibiae (Lanzhou Veterinary Research Institute, CAAS). Cells were cultured in α-MEM with 10% FBS (Gibco, Waltham, MA, USA) containing M-CSF (20 ng/mL; ABclonal, Wuhan, China). Osteoclast differentiation was induced on day 4 by adding RANKL protein (40 ng/mL; ABclonal, Wuhan, China). Murine osteoblastic MC3T3 cells, osteocytic MLO cells, and macrophage RAW 264.7 cells were cultured in α-MEM with 10% FBS or DMEM with 10% FBS (Gibco, Waltham, MA, USA) at 37 °C with 5% CO_2_.

### 2.8 Infection and intracellular survival

BMMs, osteoblastic MC3T3 cells, osteocytic MLO cells, and RAW264.7 macrophages were infected with *Brucella* strains at multiplicities of infection (MOI) of 200:1, 400:1, or 1000:1 for 4 h. Extracellular bacteria were killed with gentamicin (50 μg/mL for 1 h), and intracellular survival was assessed in medium containing gentamicin (25 μg/mL). Cells were lysed with 0.5% Triton X-100 at 0, 24, 48, and 72 h post-infection. Lysates were serially diluted and plated on TSA for CFU enumeration.

### 2.9 Immunofluorescence

Cells were blocked with Immunol Staining Blocking Buffer (Beyotime, Shanghai, China) and then incubated overnight at 4 °C with in-house goat anti-*Brucella* polyclonal and rabbit anti-*β*-catenin monoclonal (Proteintech, Wuhan, China) primary antibodies diluted 1:200 in primary antibody dilution buffer (Beyotime, Shanghai, China). After washing, cells were incubated with AF647-conjugated Donkey Anti-Goat IgG (1:500; Absmart, Nanjing, China) and YSFluor™ 488-conjugated Donkey Anti-Rabbit IgG (1:200; Yeasen, Shanghai, China) in secondary antibody dilution buffer (Beyotime, Shanghai, China) for 1 h at room temperature in the dark. Nuclei were counterstained with DAPI (5 μg/mL; ABclonal, Wuhan, China). Coverslips (Solarbio, Beijing, China) were mounted, and samples were imaged using a Zeiss LSM 980 with Airyscan 2 super-resolution system (Carl Zeiss AG, Oberkochen, Germany).

### 2.10 Hematoxylin and eosin staining and immunohistochemistry staining

Tissue samples were fixed in 10% neutral buffered formalin, processed, embedded in paraffin, and sectioned. For hematoxylin and eosin (H&E) staining, sections were stained with Harris hematoxylin and eosin Y. For immunohistochemistry (IHC) staining, samples were subjected to deparaffinization and antigen retrieval (citrate buffer, pH 6.0), and endogenous peroxidase was blocked (3% H_2_O_2_/methanol). Non-specific binding was blocked with 5% BSA. Sections were incubated overnight at 4 °C with in-house mouse anti-*Brucella* polyclonal antibody (1:200 in PBS/1% BSA), followed by HRP-conjugated AffiniPure Goat Anti-Mouse IgG (1:500; Yeasen, Shanghai, China) for 1 h at room temperature. Signals were developed with diaminobenzidine and nuclei were counterstained with hematoxylin. Slides were dehydrated, cleared, and mounted (Permount, Fisher Scientific, Waltham, MA, USA).

### 2.11 Bone resorption assay

Primary BMMs were seeded onto sterile bovine cortical bone slices and were differentiated into osteoclasts with M-CSF and RANKL for 14 days. Slices were treated with 10% sodium hypochlorite, washed, dehydrated, critical-point dried, and sputter-coated with gold. Resorption pits were visualized by field emission scanning electron microscopy (SEM) with the SU8010 ultra-high resolution scanning electron microscope (Hitachi High-Tech, Tokyo, Japan). Resorbed area percentage was quantified using ImageJ.

### 2.12 Osteoclast differentiation

Primary BMMs were differentiated with M-CSF and RANKL for up to 16 days. Cells were stained for tartrate-resistant acid phosphatase (TRAP) activity using a TRAP Stain Kit (Nanjing Jiancheng Bioengineering Institute, Nanjing, China). TRAP-positive multinucleated cells (≥3 nuclei) were counted as mature osteoclasts, and density was expressed as cells per field.

### 2.13 Quantitative RT-PCR

Total RNA was extracted from cells using RNAex Pro RNA Extraction Reagent (Accurate Biology, Changsha, China), quantified, and reverse transcribed using the Evo M-MLV Reverse Transcription Premix Kit (Accurate Biology, Changsha, China). Quantitative RT-PCR was performed on an ABI 7500 system using the One-Step RT-qPCR Kit (Accurate Biology, Changsha, China) and specific primers (**Supplementary Table S1**). Relative gene expression was calculated using the 2−ΔΔCt method with β-actin as reference.

### 2.14 Statistical analysis

Experiments were performed in triplicate. Data are mean ± SD. SPSS 22 was used. Unpaired two-tailed *t*-tests (Welch’s correction) were used for pairwise comparisons. One-way ANOVA (Tukey’s HSD) was used for multi-group comparisons. Values were deemed statistically significant when *P* < 0.05.

## 3 Results

### 3.1 Three isolates of *B. melitensis* biovar 3 were obtained from lesioned bone tissue of patients with *Brucella* spondylitis in Gansu Province, China

**Figure 1:**
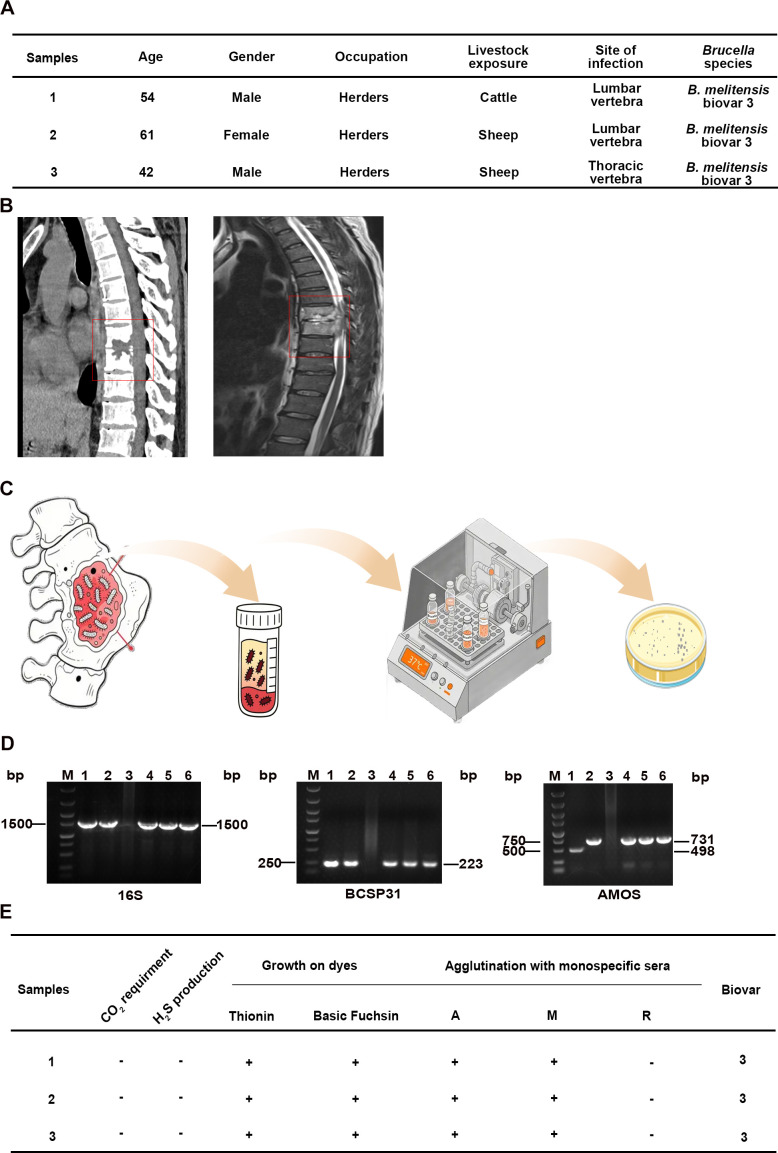
Isolation and identification of *Brucella* from bone tissue specimens of patients with brucellar arthritis. **(A)** Demographic characteristics of patients with brucellar arthritis. **(B)** Computed tomography and magnetic resonance imaging of patients with brucellar spondylitis. **(C)** Bacterial isolation procedure from lesioned bone tissue specimens of brucellar arthritis patients. **(D)** Identification of *Brucella* by 16S rRNA, BCSP31, and AMOS-PCR assays. 16S rRNA and BCSP31 indicate *Brucella* genus, whereas AMOS-PCR determines *B. abortus* or *B. melitensis*. M, DNA molecular weight marker; 1, *B. abortus*-positive control strain; 2, *B. melitensis*-positive control strain; 3, negative control; 4, 5, and 6, three clinical isolates. **(E)** Comprehensive biotyping assays for *Brucella* identification: CO_2_ requirement, H_2_S production, growth on thionin and basic fuchsin plates, and monospecific serum agglutination tests.

To acquire clinical *Brucella* strains from patients with brucellar spondylitis and determine their species, we collected bone tissue specimens along with demographic and clinical characteristics from three patients diagnosed with brucellar arthritis in Gansu, China (**Figure 1A**). Imaging revealed *Brucella*-induced osseous defects and significant structural destruction in the spinal vertebrae of the patients (**Figure 1B**). Lesioned bone tissues were collected which yielded three *Brucella* strains for subsequent analysis (**Figure 1C**). Species identification via 16S rRNA, BCSP31, and AMOS-PCR indicated that all three isolates were *B. melitensis* (**Figure 1D**). Further biotyping assays, including CO_2_ requirement, H_2_S production, growth on thionin- or basic fuchsin-containing TSA, and monospecific A/M/R serum agglutination, identified these isolates as *B. melitensis* biovar 3 (**Figure 1E**). Taken together with existing literature, our findings lead us to hypothesize that *B. melitensis* biovar 3 may be one of the primary pathogenic strains responsible for *Brucella*-induced arthritis (Zhu et al., 2024).

### 3.2 Whole genome sequencing-based molecular epidemiology of *B. melitensis* arthritis isolates

**Figure 2:**
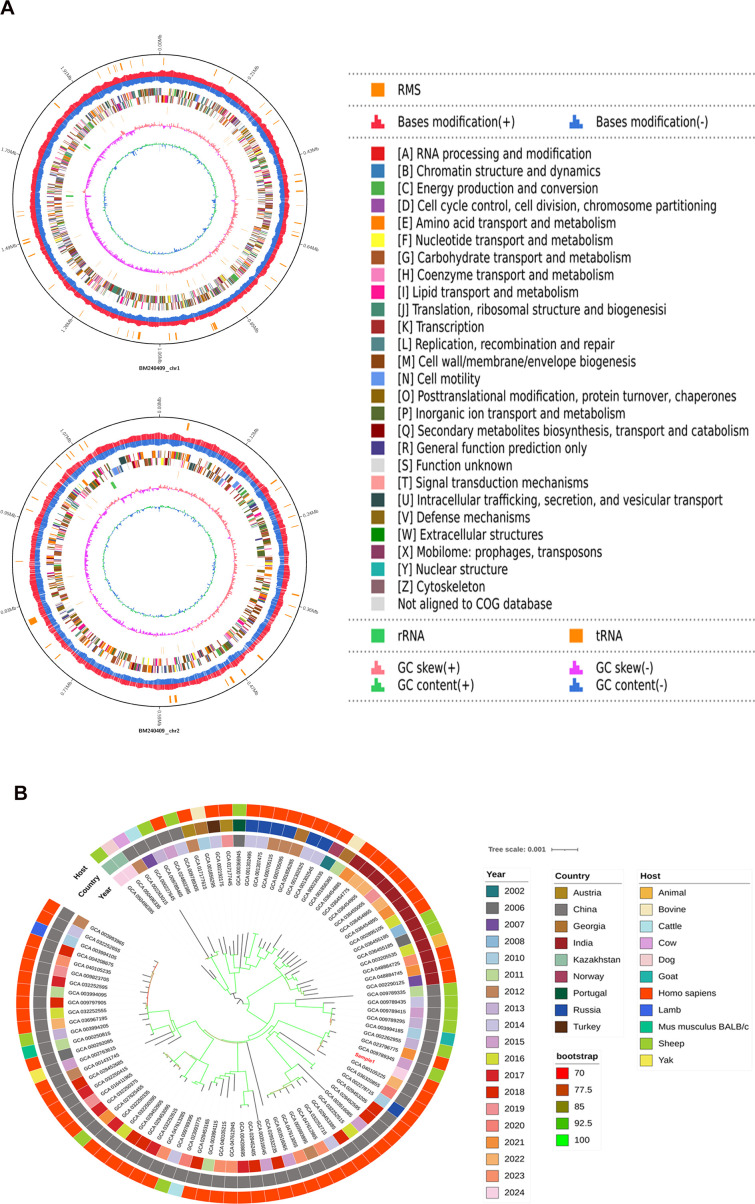
Visualization of whole genome sequencing results and maximum likelihood tree based on wgSNP analysis. **(A)** Circular genome map displaying whole genome sequencing results. **(B)** Maximum likelihood tree constructed from wgSNP comparisons between clinical isolates and publicly available *B. melitensis* strains in NCBI annotated with isolation location, date, and host (color-coded).

To characterize the genomic features of this clinical isolate, the genomic and epidemiological features of one of the *B. melitensis* biovar 3 isolates were analyzed by whole genome sequencing. Results were visualized using a circular genome map which integrated multiple features including GC content, GC skew, tRNA and rRNA genes, Clusters of Orthologous Genes annotations, and genes related to base modification and restriction-modification systems (**Figure 2A**).

A maximum-likelihood phylogenetic tree was constructed using whole genome sequencing data from clinical isolates and NCBI reference strains for epidemiological analysis (**Supplementary Figure S1**). The tree, in which bootstrap values are indicated for each branch, included globally distributed strains. The clinical isolate from Gansu clustered closely with strains from diverse Chinese regions, including Yangzhou, Hebei, and Inner Mongolia, which suggests wide dissemination of this lineage. Notably, most strains in the cluster were human-derived, with a minority originating from sheep, which indicates potential sheep-to-human transmission that identifies these strains as putative epidemic strains of human brucellosis (**Figure 2B**).

### 3.3 *B. melitensis* biovar 3 clinical isolates are hypervirulent yet do not induce splenomegaly during early infection in mice

**Figure 3:**
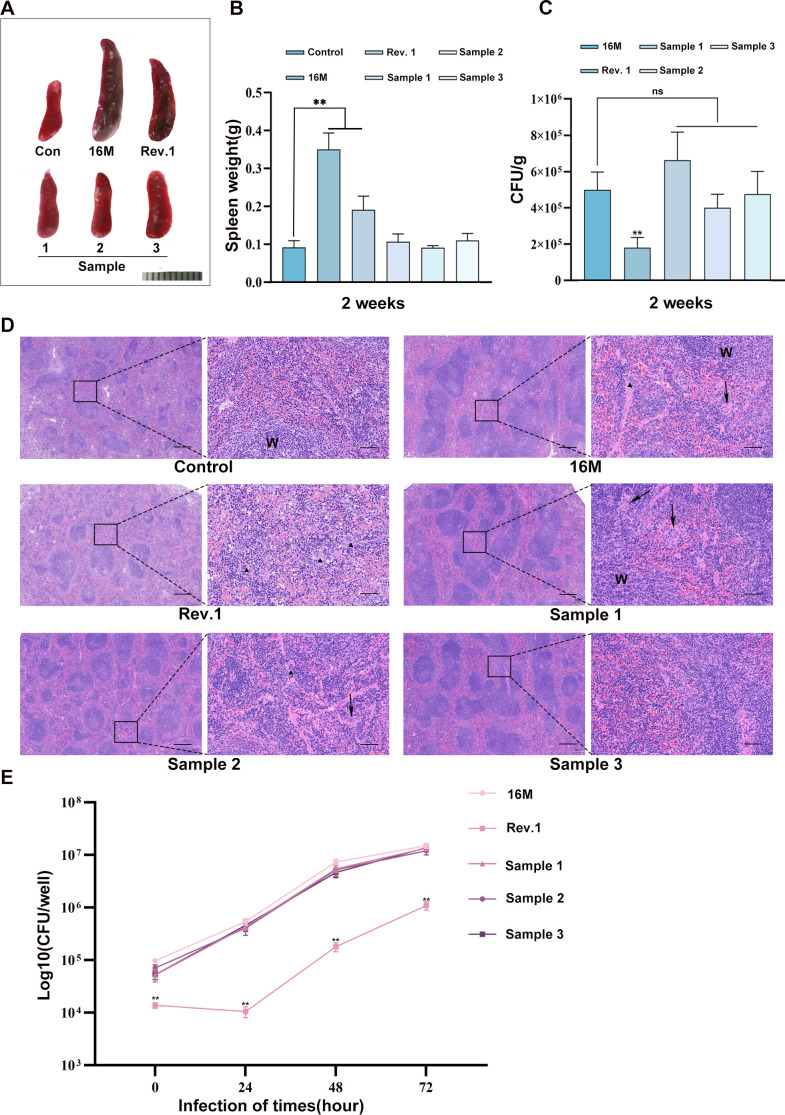
Virulence assessment of *B. melitensis* biovar 3 clinical isolates *in vitro* and *in vivo*. **(A)** Spleens of mice infected with different *B. melitensis* clinical isolates, virulent strain 16M, and vaccine strain Rev.1 for two weeks. Scale bars: 1 cm. **(B)** Spleen weight two weeks post-infection. Data are presented as mean ± SD. One-way ANOVA followed by Tukey’s multiple comparison test; **P < 0.01. **(C)** Bacterial burden expressed as CFU/gram of spleen tissue. Data are presented as mean ± SD. One-way ANOVA followed by Tukey’s multiple comparison test; ns, *P*>0.05, **P < 0.01. **(D)** Representative micrographs of splenic histopathology at four weeks post-infection. Scale bars: 400 μm and 60 μm. W, white pulp; arrowheads, granulomas; triangles, multinucleated giant cells. **(E)** Intracellular bacterial CFU counts in RAW264.7 infected with strains 16M, Rev.1, and three clinical isolates at an MOI of 400 across different time points. Data are presented as mean ± SD. One-way ANOVA followed by Tukey’s multiple comparison test; **P < 0.01.

To assess the virulence of the clinical *Brucella* isolate, the virulence of the *B. melitensis* biovar 3 clinical isolates was evaluated using a BALB/c mouse infection model. Bacterial burdens in spleens were quantified by plate counting two weeks post-intraperitoneal challenge, and splenomegaly was assessed through spleen weight measurement. Mice infected with either virulent *B. melitensis* 16M or the live attenuated Rev.1 vaccine strain exhibited significant splenomegaly compared to control animals administered only PBS, with more pronounced spleen weight increase in animals infected with the 16M strain than with the Rev.1 strain. These findings align with the established virulence hierarchy in which the more pathogenic *B. melitensis* 16M induces a stronger immune response than Rev.1. In contrast, intraperitoneal inoculation with any of the three clinical isolates failed to induce significant splenomegaly in BALB/c mice, with no statistically significant difference in spleen weight compared to PBS-injected control mice (**Figures 3A, B**). Bacterial burden in mouse spleens was assessed further by enumeration of bacterial loads in spleen homogenates. Values were determined by plate counting and calculating CFU/g of spleen tissue. No significant difference in bacterial density was observed following infection with the 16M strain or clinical isolates, although both showed significantly higher values than infection with the Rev.1 strain (**Figure 3C**). Histopathological examination demonstrated that all infected mice developed splenic inflammatory pathology that featured white pulp expansion and indistinct marginal zones compared to uninfected animals. However, in contrast to the pronounced white pulp hyperplasia with effaced marginal zones observed in both 16M- and Rev.1-infected animals, mice administered the clinical isolates exhibited only moderate white pulp expansion with relatively well-defined tissue demarcation (**Figure 3D**). These data indicate that bacterial burden *per se* is insufficient to trigger splenic inflammation. During the early phase of infection, clinical isolates exhibit attenuated pro-inflammatory signaling and leukocyte recruitment compared to reference strains, including the attenuated Rev.1. This feature may contribute to the ability of *B. melitensis* biovar 3 to evade initial host clearance mechanisms.

The virulence of clinical isolates *in vitro* was evaluated further by intracellular survival assays using RAW264.7 macrophages. The clinical isolates and *B. melitensis* 16M maintained comparable loads at all time points examined, with both showing significantly higher intracellular counts than Rev.1 (**Figure 3E**). Collectively, these findings indicate that the clinical isolates exhibit virulence comparable to the *B. melitensis* 16M both *in vivo* and *in vitro* but. unlike the 16M strain, fail to induce splenomegaly during early infection in mice.

### 3.4 *B. melitensis* biovar 3 clinical isolates colonize both mice and human bone tissue

**Figure 4:**
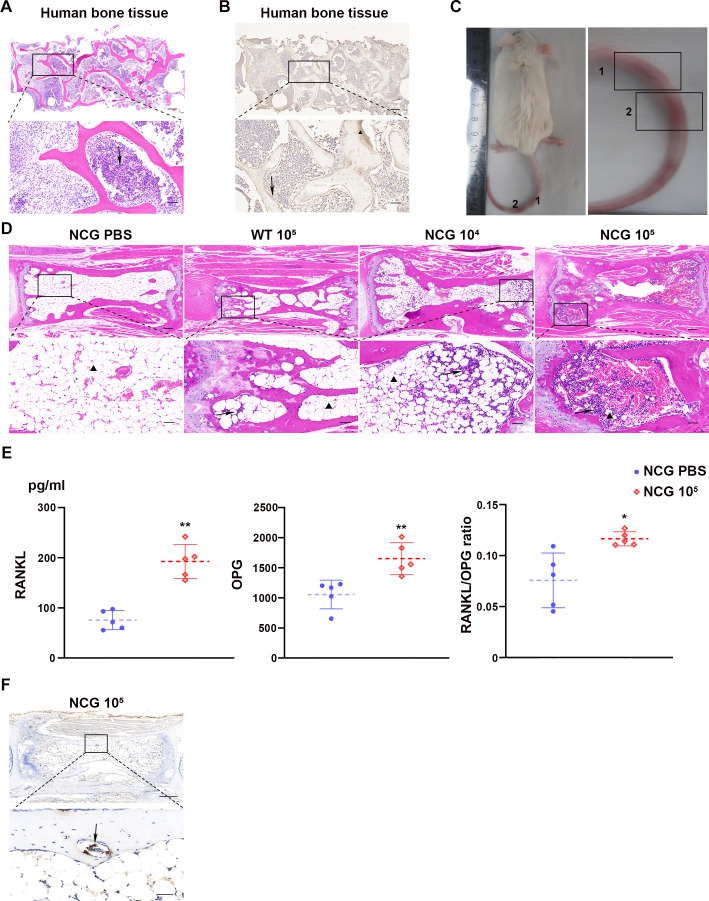
*B. melitensis* biovar 3 colonization in human and murine bone tissues, with induction of tail vertebrae swelling and erythema in NCG mice. **(A)** H&E staining of lesioned vertebral bone tissue in human. Scale bars: 200 μm and 50 μm. Arrowheads indicate an area of inflammatory cell infiltration accompanied by severe trabecular bone destruction. **(B)** Representative immunohistochemical micrographs of *Brucella* in human vertebral lesions. Scale bars: 200 μm and 50 μm. Arrows indicate osteoclasts; triangles denote antigen accumulation. **(C)** NCG mouse model of *Brucella*-induced osteomyelitis. 1 and 2 indicate tail vertebral lesions with swelling. **(D)** Representative histopathological micrographs of mouse caudal vertebrae at eight weeks post-infection. NCG PBS, NCG mice receiving 200 μL of PBS via intraperitoneal (i.p.) injection; NCG 10^4, NCG mice challenged i.p. with 1×10^4 CFU of *Brucella* in 200 μL; NCG 10^5, NCG mice challenged i.p. with 1×10^5 CFU of *Brucella* in 200 μL; WT 10^6, wild-type (WT) mice challenged i.p. with 1×10^6 CFU of *Brucella* in 200 μL. Scale bars: 200 μm and 50 μm. Triangles indicate trabecular bone; arrowheads indicate areas of inflammatory cell infiltration accompanied by severe trabecular bone destruction. **(E)** Blood was obtained from the fundus vein of NCG PBS and NCG 10^5^ mice. The concentrations of RANKL and OPG were determined by ELISA in the serum. Each data point was from an individual mouse and means were indicated by dashed horizontal lines. Data are presented as mean ± SD. Unpaired two-tailed *t*-test with Welch’s correction; **P < 0.01, *P < 0.05. **(F)** Immunohistochemical analysis of *Brucella* antigens in NCG mouse tail vertebrae. Arrows indicate *Brucella* antigen accumulation in osteoclasts. Scale bars: 400 μm and 40 μm.

Histopathological H&E staining of human vertebral bone specimens was performed to investigate *B. melitensis* biovar 3 colonization in human and murine bone tissues and to assess pathogenically-induced osseous defects. This analysis revealed severe *Brucella* infection-induced structural damage that was characterized by distorted and fractured trabeculae with markedly widened and irregular intertrabecular spaces (**Figure 4A**). Dense inflammatory infiltrates were observed within the disrupted bone matrix and marrow cavities, forming distinct chronic inflammatory foci. IHC analysis of human vertebral tissues further confirmed that *Brucella* not only colonizes bone tissue but also proliferates within various types of bone cells, with notably high concentrations of antigen accumulation observed particularly in osteoclasts (**Figure 4B**).

A murine model using immunodeficient NCG mice was established based on published protocols to facilitate studies on *Brucella*-induced osteomyelitis (Al Dahouk et al., 2003). NCG mice developed tail swelling and curvature approximately 8 weeks post-inoculation *B. melitensis* biovar 3 clinical isolates, which confirmed successful model establishment (**Figure 4C**). Histopathological examination showed that all mice inoculated with the *B. melitensis* clinical isolates exhibited MOI-dependent severe tail inflammation, featuring extensive macrophage and neutrophil infiltration in bone marrow, enhanced bone resorption, and intervertebral disc erosion with fibrosis. In contrast, wild-type mice displayed milder pathological damage and inflammatory responses (**Figure 4D**) which demonstrates that brucellar arthritis cannot be modeled effectively in immunocompetent wild-type mice.

We subsequently quantified serum levels of RANKL and OPG by ELISA in a mouse model of *Brucella*-induced arthritis and calculated their ratio. The results demonstrated a significant increase in the RANKL/OPG ratio in NCG 10^5^-infected mice compared to the control group, indicating enhanced osteoclast-mediated bone resorption and confirming successful model establishment. Notably, we observed a concurrent increase in OPG, a marker of osteoblastic activity, although its level of upregulation was substantially lower than that of RANKL (**Figure 4E**). This finding suggests that while the osteogenic process is activated in response to *Brucella* infection, it is insufficient to compensate for the more pronounced increase in bone resorptive activity.

IHC analysis using both bright-field and fluorescence microscopy determined *Brucella* distribution in bone tissues. Infected mouse caudal vertebrae exhibited intense immunostaining, with *Brucella* antigens widely distributed throughout sections. Bacterial antigens localized primarily to marrow cavities, subchondral bone, and bone resorption areas. Osteoclasts showed the highest *Brucella* antigen concentration among various bone cell types (**Figure 4F**).

### 3.5 *B. melitensis* clinical isolates infect all bone cell types but exhibit strongest tropism for osteoclasts

**Figure 5:**
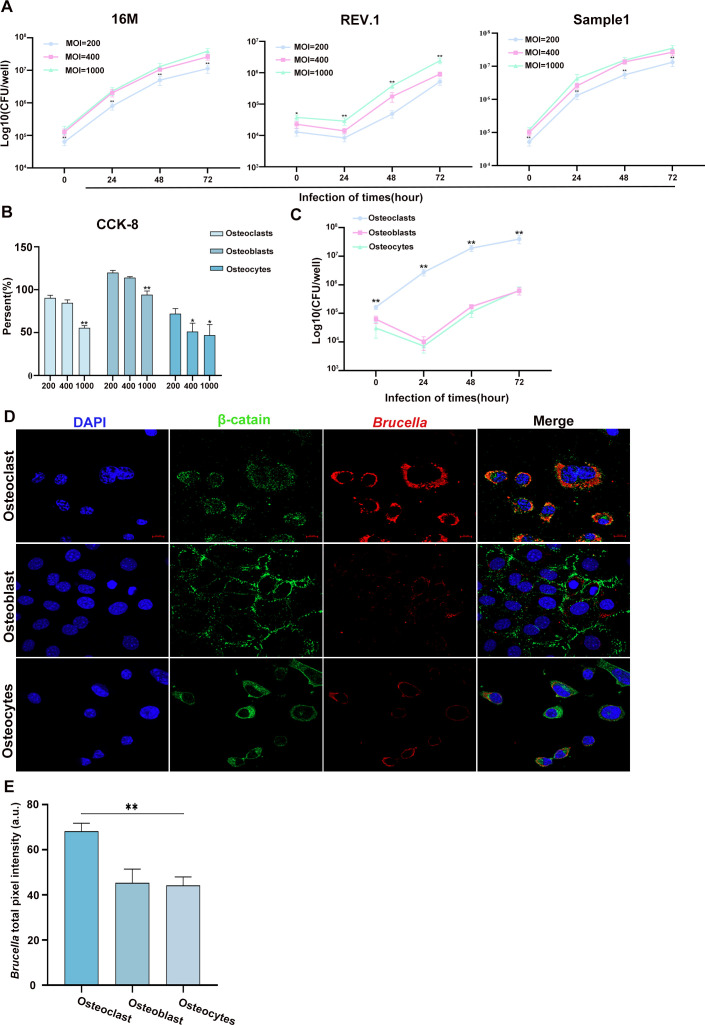
*B. melitensis* biovar 3 invades all types of bone tissue cells with MOI-dependent effects on osteocyte viability and exhibits stronger tropism for osteoclasts compared to osteoblasts and osteocytes. **(A)** Intracellular bacterial loads (CFU) in primary monocytes infected with clinical isolates and reference virulent and attenuated strains at different MOIs and time points. Data represent mean ± SD. One-way ANOVA with Tukey’s *post-hoc* test; *P < 0.05, **P < 0.01. **(B)** Viability of different bone tissue cells under various MOI infection conditions. Data represent mean ± SD. One-way ANOVA with Tukey’s *post-hoc* test; *P < 0.05, **P < 0.01. **(C)** Intracellular CFU counts in different bone tissue cells at MOI = 400 across time points. Data represent mean ± SD. One-way ANOVA with Tukey’s *post-hoc* test; **P < 0.01. **(D)** Representative immunofluorescence micrographs of various cells 72 h post-infection at MOI = 400. Scale bar: 10μm. Blue: nuclei; red: *Brucella*; green: β-catenin. **(E)** Quantitative analysis of *Brucella* red fluorescence intensity. Data represent mean ± SD. One-way ANOVA with Tukey’s *post-hoc* test; **P < 0.01.

The tropism of clinical *B. melitensis* isolates for different bone tissue cell types was evaluated further with *in vitro* intracellular survival assays using diverse bone-derived cells. CCK-8 cell viability assays and preliminary intracellular survival tests initially were performed to determine the optimal MOI. No significant differences in intracellular bacterial loads were observed between MOI = 400 and MOI = 1000, except for infection with Rev.1 (**Figure 5A**) for which cell viability at MOI = 400 was significantly higher than that at MOI = 1000 (**Figure 5B**). Therefore, MOI = 400 was selected for subsequent bacterial challenges. Further intracellular survival assays demonstrated that clinical *Brucella* isolates exhibited significantly stronger tropism for osteoclasts than for osteoblasts and osteocytes at all time points examined which suggests that *B. melitensis* biovar 3 preferentially infects osteoclasts (**Figure 5C**).

The enhanced tropism of *B. melitensis* for osteoclasts was examined further by employing immunofluorescence imaging to visualize bacterial distribution among different bone cell types. This analysis revealed more abundant and densely clustered red fluorescence signals in osteoclasts, which is indicative of *Brucella* infection (**Figure 5D**). Fluorescence intensity was measured and analyzed for quantitative verification which demonstrated significantly higher *Brucella*-associated red fluorescence intensity in osteoclasts compared to osteoblasts and osteocytes (**Figure 5E**). These results indicate clearly that *B. melitensis* biovar 3 exhibits the strongest tropism for osteoclasts among bone tissue cells.

### 3.6 Supernatants from *B. melitensis-*infected osteoblasts and osteocytes promote early osteoclast differentiation but do not affect late-stage fusion and apoptosis

**Figure 6:**
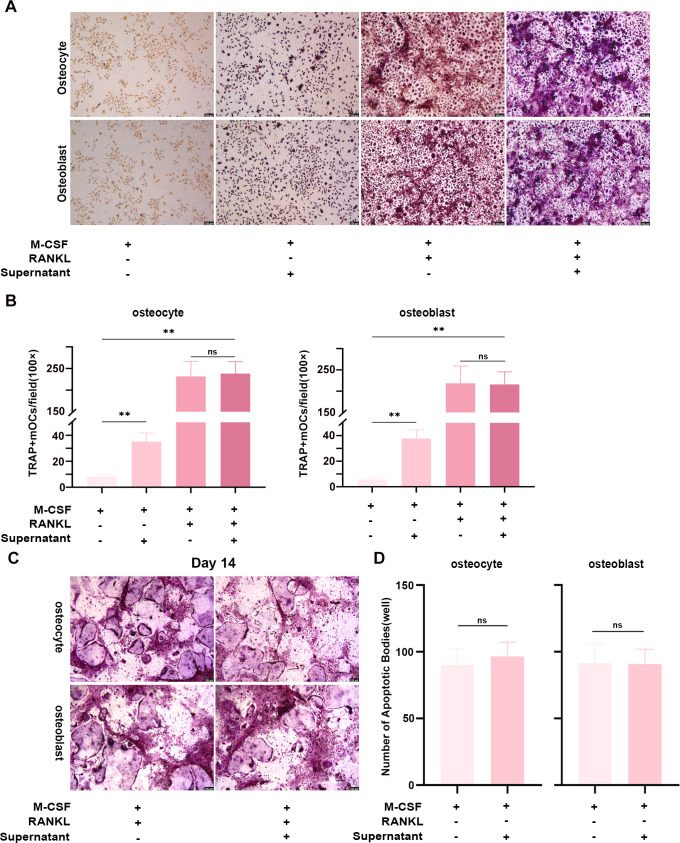
Differentiation of BMMs into multinucleated mature osteoclasts in the presence of conditioned medium from *B. melitensis-*infected osteocytes and osteoblasts, with parallel assessment of apoptosis- and autophagy-related marker genes in the infected osteocytes and osteoblasts. **(A)** Bright-field micrographs of BMMs differentiation after six days of culture with M-CSF (constant) with and without RANKL and infection-conditioned supernatants. Scale bar: 100 μm. **(B)** Quantification of mature osteoclasts (>3 nuclei) from **(A)**. One-way ANOVA with Tukey’s *post-hoc* test; ns, *P*>0.05, **P < 0.01. **(C)** Bright-field micrographs of BMMs cultured for 14 days with M-CSF (constant) in the presence or absence of RANKL and infection-conditioned supernatants. Scale bar: 100 μm. **(D)** Quantification of apoptotic vesicles from **(C)**. Data represent mean ± SD. Unpaired two-tailed *t*-test with Welch’s correction; ns, *P*>0.05.

Since *Brucella* infection disrupts osteocyte and osteoblast homeostasis and stimulates the release of pro-inflammatory factors that promote osteoclast differentiation (Moley et al., 2023; Pesce Viglietti et al., 2019; Scian et al., 2011b), we sought to dissect its direct effects independent of these paracrine signals. Thus, primary BMMs were cultured for six days with conditioned media from osteocytes and osteoblasts infected with clinical *B. melitensis* isolates, using appropriate controls to assess stage-specific osteoclast differentiation and late-stage apoptosis. Results showed that conditioned media from infected osteocytes and osteoblasts promoted early-stage osteoclast differentiation and supported BMMs commitment to osteoclast precursors. However, this pro-differentiation effect was less pronounced than that induced by exogenous RANKL. Furthermore, when infection-conditioned supernatants were combined with RANKL, no significant difference in mature osteoclast numbers was observed. These findings suggest that the early pro-differentiation activity may be mediated by low concentrations of RANKL released from *B. melitensis*-infected osteocytes and osteoblasts, and that such endogenous RANKL release does not exert significant additive effects when background RANKL concentrations are already sufficient (**Figures 6A, B**).

BMMs were cultured for 14 days in medium containing M-CSF and RANKL and with or without *B. melitensis* infection-conditioned supernatants from osteocytes and osteoblasts to determine whether these extracts affect late-stage osteoclast differentiation, fusion, and apoptosis. All cultures exhibited abundant apoptotic vesicles and large acellular areas that contained cellular debris but no intact cells (**Figure 6C**). No significant difference in apoptotic vesicle counts were observed regardless of whether supernatants were derived from infected osteocytes or osteoblasts (**Figure 6D**). These results demonstrate that *B. melitensis* infection-conditioned supernatants exert no significant effect on late-stage osteoclast differentiation, fusion, or apoptosis.

Autophagy- and apoptosis-related gene expression in osteoblasts and osteocytes infected with clinical *B. melitensis* isolates was quantitated by RT-qPCR. The isolates showed trending effects on autophagy and apoptosis gene expression in osteocytes, but not in osteoblasts, which suggest that osteoblasts may be more resistant to *Brucella* invasion than osteocytes (**Supplementary Figure S2**).

### 3.7 *B. melitensis* biovar 3 influences the differentiation and apoptosis of osteoclasts and bone resorption capacity

**Figure 7:**
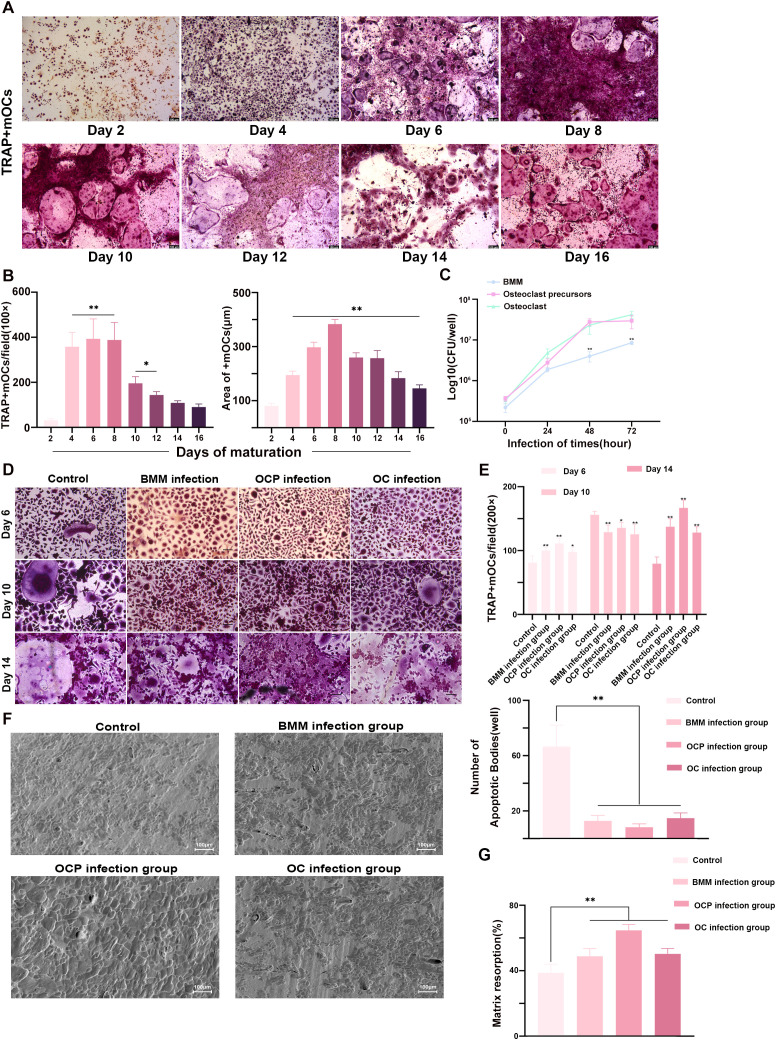
*B. melitensis* invasion of osteoclasts alters differentiation, fusion, and late-stage apoptosis, ultimately affecting long-term bone resorption capacity. **(A)** Bright-field micrographs of BMM-derived osteoclast differentiation in 10% complete medium containing M-CSF (20 ng/ml) and RANKL (40 ng/ml) which illustrate morphological changes at different time points. Scale bar: 100 μm. **(B)** Quantitative analysis of mature osteoclast numbers (≥3 nuclei) and coverage area from **(A)**. Data represent mean ± SD. One-way ANOVA with Tukey’s *post-hoc* test; *P < 0.05, **P < 0.01. **(C)** Intracellular CFU at day 6 post-infection at different osteoclast differentiation stages to determine optimal infection timing. Data represent mean ± SD. One-way ANOVA with Tukey’s *post-hoc* test; **P < 0.01. **(D)** TRAP staining at early, mid, and late maturation stages to assess infection effects on terminal differentiation. Scale bar: 50 μm. **(E)** Mature osteoclast enumeration (≥3 nuclei) across maturation phases showing biphasic trends, i.e., initial increase followed by decrease, with late-stage apoptotic vesicle quantification revealing significantly higher counts in controls. Data represent mean ± SD. One-way ANOVA with Tukey’s *post-hoc* test; *P < 0.05, **P < 0.01. **(F)** SEM micrographs of resorption pits on bovine cortical bone slices after 14 days of osteoclast differentiation. BMMs were plated on bone slices and infected at different differentiation stages prior to SEM analysis. Scale bar: 100 μm. **(G)** Quantification of resorption pit areas. Data represent mean ± SD. One-way ANOVA with Tukey’s *post-hoc* test; **P < 0.01.

The preceding experiments established that osteoclasts are the preferred target cells among bone tissue cell populations for *B. melitensis* biovar 3, and that factors secreted by infected osteoblasts and osteocytes promote early-stage osteoclast differentiation without affecting long-term survival. Therefore, we investigated whether *B. melitensis* directly modulates osteoclast differentiation and survival, thereby contributing to pathological bone destruction in *Brucella*-induced osteomyelitis. The temporal progression of BMMs differentiation into osteoclasts was delineated first by inducing continuous differentiation for 16 days, with samples collected every two days for TRAP staining analysis (**Figure 7A**). Cultures at day 2 predominantly contained osteoclast precursors with few mature osteoclasts, whereas mature osteoclast numbers increased dramatically by day 4 (**Figure 7B**). Therefore, the period before day 4 was defined as the precursor stage and subsequent differentiation from day 4 onward was defined as the maturation stage. Cells subsequently were challenged at three stages to identify optimal infection timepoints: BMM stage, precursor stage (day 2), and maturation stage (day 4), followed by quantification of intracellular bacteria. The highest loads occurred when *B. melitensis* infection was initiated at either the precursor or maturation stage both of which significantly exceeded intracellular loads from BMM-stage infections (**Figure 7C**).

The temporal effects of *Brucella* infection on osteoclast differentiation were assessed by TRAP staining of osteoclasts at different time points. Staining at day 6 revealed that the precursor-stage infection group yielded the highest number of mature osteoclasts compared to controls, although these cells predominantly were newly-matured, roundish cells with approximately three nuclei, whereas the uninfected control group exhibited deeply stained, fully mature osteoclasts containing >20 nuclei (**Figure 7D**). Apoptotic vesicle quantification at day 14 showed significantly higher counts in uninfected control group compared to the *B. melitensis* clinical isolate-infected group (**Figure 7E**). These results suggest that *B. melitensis* biovar 3 promotes early osteoclast fusion, but subsequently inhibits further fusion or differentiation and ultimately prolongs osteoclast survival and increases cell numbers.

The effects of *B. melitensis* infection on bone resorption were examined using BMM cells infected at different time points. These cells were harvested after 14 days of differentiation for SEM evaluation of resorption pits on bovine cortical bone slices. All cultures formed circular resorption pits of varying sizes. However, *Brucella*-infected cells exhibited larger resorption areas compared to uninfected controls, with the precursor-stage infection group showing the most extensive pit formation (**Figure 7F**). Statistical analysis confirmed significant differences in resorption areas between infected cultures and uninfected controls and verified that precursor-stage infection had the most pronounced effect (**Figure 7G**). These findings demonstrate that the optimal infection window occurs during the osteoclast precursor stage, and that clinical *B. melitensis* biovar 3 isolates promote early osteoclast differentiation while suppressing subsequent fusion and differentiation. This dual regulatory mechanism extends osteoclast lifespan, enhances functional duration and population accumulation, and ultimately leads to increased pathological bone resorption.

## 4 Discussion

Bacteria of the genus *Brucella* are significant human and veterinary pathogens, and represent one of the major causative agents of human osteomyelitis. A comprehensive understanding of the mechanisms by which *Brucella* induces and promotes osteomyelitis is crucial for developing improved prevention and treatment strategies. Although osteoclasts are the sole bone-resorbing cells in osseous tissue, the interactions between *Brucella*, particularly clinically isolated strains, and osteoclasts remain poorly characterized. Our study provides compelling evidence that *B. melitensis* biovar 3 represents a distinct hypervirulent strain that is capable of achieving splenic bacterial loads equivalent to the virulent *B, melitensis* 16M strain, but without inducing splenomegaly during early stage in mice. This pathogen exhibits a distinct tropism for osteoclasts, enhancing early differentiation while suppressing differentiation and fusion at intermediate and late stages. Consequently, *B. melitensis* biovar 3 disrupts late-stage programmed apoptosis in osteoclasts which ultimately promotes osteoclast accumulation, exacerbates bone resorption, and induces osseous defects. These findings suggest that *B. melitensis* biovar 3 appears to be a leading epidemic strain likely responsible for human brucellar arthritis in Gansu, China.

Forty-two studies have reported *Brucella* isolates across Asia as of 2022. Although both *B. abortus* and *B. melitensis* are recognized as the two predominant species in this region, analysis of 24 human-derived isolation studies revealed exclusively *B. melitensis* or *B. melitensis*-containing strain collections (Ali et al., 2024). Supporting this epidemiological pattern, a nationwide study in Turkey (2018-2021) identified *B. melitensis* in 98.7% of 74 human clinical isolates, with only 1.3% being *B. abortus* (Özmen et al., 2023). All characterized *B. melitensis* strains in northwest China, including those from animal reservoirs, belong to biovar 3 (Cao et al., 2025). Similarly, all 28 human clinical isolates from Xinjiang, China were identified as *B. melitensis* biovar 3 (Liu et al., 2025) which is consistent with findings from Inner Mongolia where *B. melitensis* exclusively was isolated from blood cultures of arthritis patients (Zhu et al., 2024). Together with our isolation of biovar 3 strains from spinal lesions of brucellar spondylitis patients in Gansu, these data collectively indicate that although both *B. melitensis* and *B. abortus* are widely distributed across Asia, *B. melitensis* biovar 3 is likely the predominant etiological agent responsible for human brucellar osteoarthritis in this region.

Numerous studies have identified *B. melitensis* biovar 3 as the predominant epidemic strain that causes human brucellosis (Zhang et al., 2021) and antibiotic susceptibility profiles of clinical *Brucella* isolates have been reported (Gültekin et al., 2021). However, the virulence characteristics of biovar 3 clinical isolates have not been investigated fully. Here, both *in vitro* and *in vivo* studies demonstrated that *B. melitensis* biovar 3 clinical isolates achieved bacterial burdens comparable to the virulent *B. melitensis* 16M strain. Both the clinical isolates and 16M strain induced nearly identical mortality kinetics in NCG mice, with deaths first occurring at approximately 2.5 months post-infection and progressing at similar rates thereafter (data not shown). Interestingly, the biovar 3 isolates failed to induce splenomegaly during early stage despite maintaining splenic bacterial burdens equivalent to strain 16M. This observation suggests a potential deficiency in virulence signals required to trigger acute inflammatory cascades in *B. melitensis* biovar 3, resulting in insufficient immune cell infiltration and consequent decoupling of bacterial load from organ swelling during early stage (Grilló et al., 2012). Consequently, the immune system fails to eliminate a sufficient number of bacteria during the early stage of infection, thereby allowing a substantial population of *Brucella* to establish infection, proliferate, and ultimately precipitate a spectrum of severe complications. This distinctive phenotype likely reflects distinct immune evasion mechanisms that may explain the propensity of biovar 3 isolates to cause chronic brucellar arthritis in humans. In addition to the anatomical particularities of osseous tissue, our findings suggest that two mechanisms may contribute to bacterial persistence: the intracellular survival of *Brucella* within inherently resistant osteoclasts and its potential failure to trigger acute inflammation. These mechanisms represent key foci for our ongoing investigations. These discoveries demonstrate that, in addition to inflammation-mediated effects on osteoclastogenesis and bone resorption (Delpino et al., 2012; Giambartolomei et al., 2012; Pesce Viglietti et al., 2016, 2019; Scian et al., 2013, 2011a, 2011b), direct *B. melitensis*-osteoclast interactions contribute substantially to the development of pathological bone defects in brucellar arthritis.

Only a single study to date has investigated the interaction between *Brucella* and osteoclasts (Khalaf et al., 2020). The analysis demonstrated that both *B. abortus* strains 2308 and S19 replicate within mature osteoclasts. Furthermore, infection with *B. abortus* 2308 suppressed osteoclast formation but neither diminished osteoclast resorptive activity nor induced apoptosis in mature osteoclasts. Certain aspects of these findings align with our results, specifically the lack of *Brucella*-induced apoptosis in osteoclasts and the suppression of osteoclast differentiation and fusion at intermediate and late stages. However, a key discrepancy exists between our findings and the previous study (Khalaf et al., 2020). Here, infection with clinical isolates at the monocyte/osteoclast precursor stage resulted in significantly enhanced bone resorption capacity after 14 days of total differentiation compared to uninfected cells with normally induced differentiation. In contrast, infection with *B. abortus* 2308 at the same differentiation stage suppressed osteoclast-mediated bone resorption (Khalaf et al., 2020). This discrepancy may stem from differences in sampling time points as well as the bacterial species used in the respective experiments, i.e., *B. melitensis* and *B. abortus*. Moreover, the current study directly addressed the clinical context by utilizing clinical *B. melitensis* isolates obtained from patients with brucellar arthritis. The virulence of these isolates was confirmed and a murine model of brucellar spondylitis was established using the isolates. Subsequent *in vitro* studies demonstrated that the main *B. melitensis* biovar 3 clinical isolate used in the study significantly impacted osteoclast differentiation. Further in-depth investigation of this clinical isolate is warranted, as *B. melitensis* biovar 3 has been identified as the predominant pathogen responsible for *Brucella*-induced arthritis (Zhu et al., 2024; Giambartolomei et al., 2017), particularly regarding its mechanistic role in driving pathological bone destruction.

In summary, clinical isolates of *B. melitensis* biovar 3 appear to exert dual regulatory effects on osteoclasts: they promote the early differentiation of osteoclast precursors into mature, tri-nucleated osteoclasts, while simultaneously suppressing further fusion of mature osteoclasts. This suppression delays osteoclast apoptosis and extends their functional lifespan. Together, these effects may represent a key pathogenic strategy that contributes to pathological bone destruction in human *Brucella*-induced arthritis. While previous studies have largely emphasized osteoclast differentiation and inflammatory pathways in bone destruction, the direct interaction between *Brucella* and osteoclasts has remained poorly understood. Our findings establish that such a direct interaction not only exacerbates bone loss but may also facilitate bacterial persistence *in vivo*. This insight underscores the importance of further investigating the molecular mechanisms by which *B. melitensis* inhibits osteoclast apoptosis and promotes osteoclast accumulation, as well as identifying the bacterial virulence factors involved. Elucidating these mechanisms will clarify the direct role of *B. melitensis* in modulating osteoclast function during the progression of *Brucella*-induced arthritic bone damage.

## Data Availability

The datasets presented in this study can be found in online repositories. The names of the repository/repositories and accession number(s) can be found in the article/**Supplementary Material**.
