# Supplementary figures and images for: Brucella melitensis clinical isolate modulates osteoclast differentiation to drive pathological bone destruction in brucellar arthritis
Source: Front Cell Infect Microbiol. 2025 Dec 12;15:1694633. doi: 10.3389/fcimb.2025.1694633 (PMC12741104; doi:10.3389/fcimb.2025.1694633)

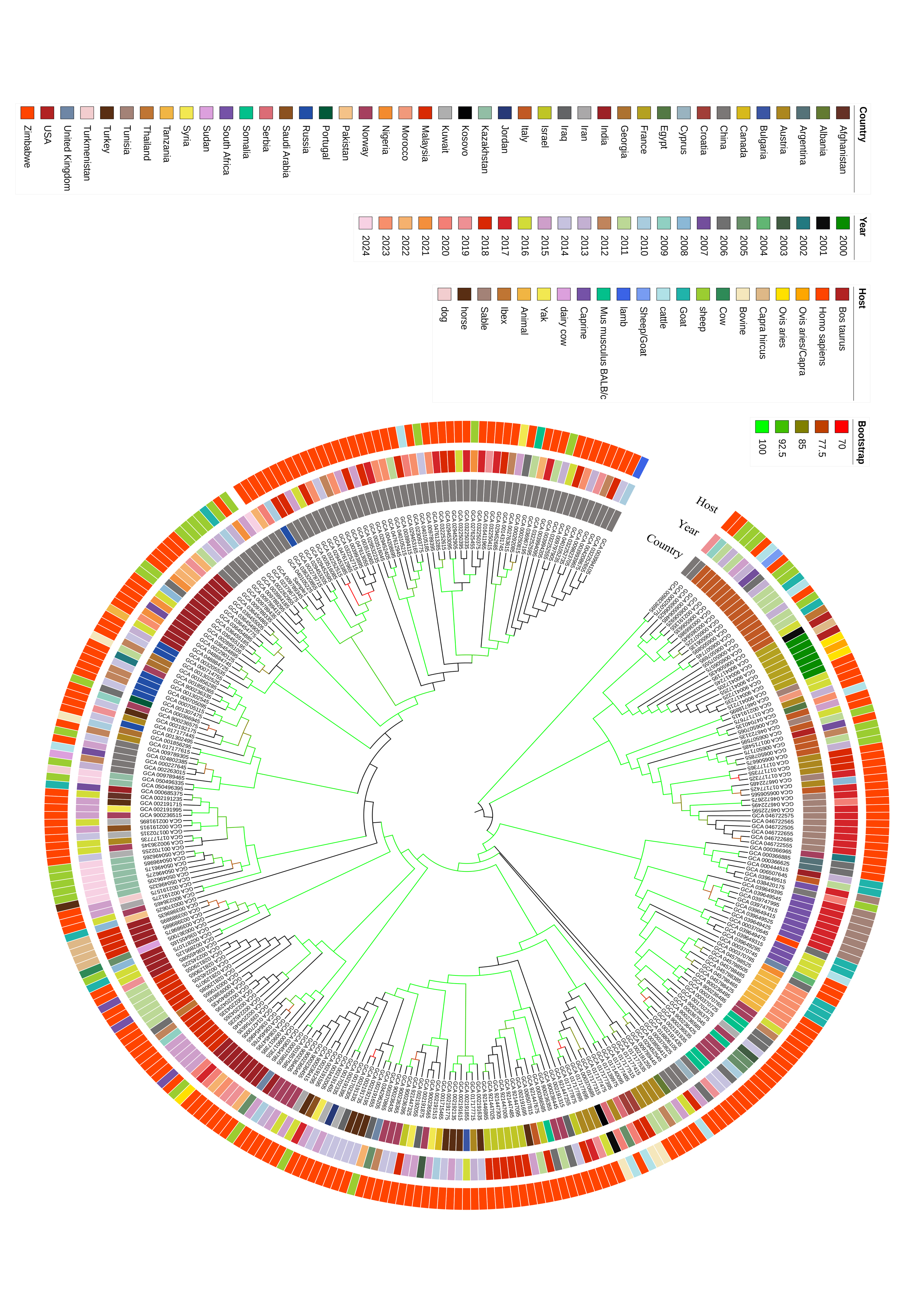

Supplement: Supplementary file 1 [file Image1.tif]

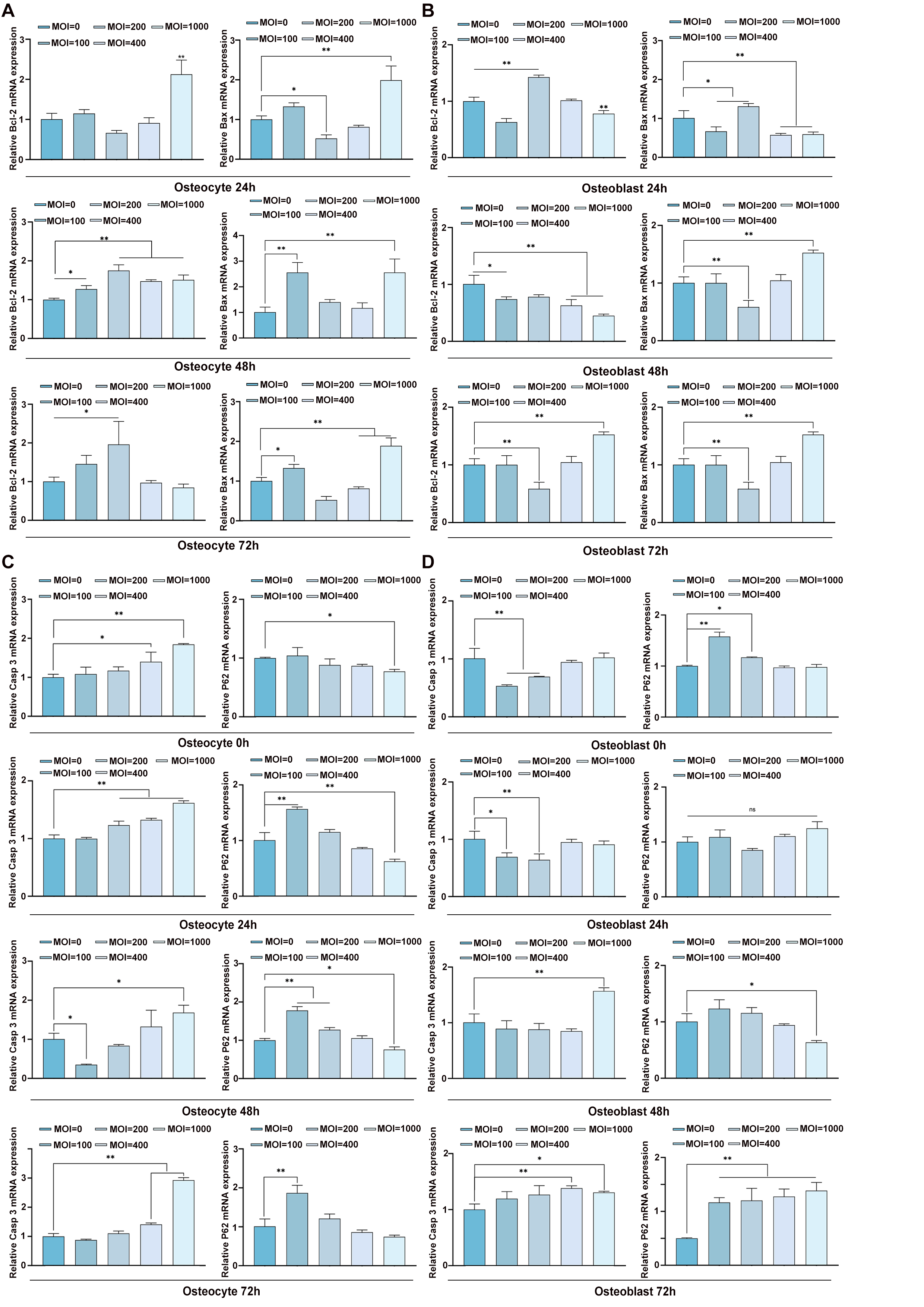

Supplement: Supplementary file 2 [file Image2.tif]
